# Association between suicide-related ideations and affective temperaments in the Japanese general adult population

**DOI:** 10.1371/journal.pone.0179952

**Published:** 2017-06-22

**Authors:** Nobuyuki Mitsui, Yukiei Nakai, Takeshi Inoue, Niki Udo, Kan Kitagawa, Yumi Wakatsuki, Rie Kameyama, Atsuhito Toyomaki, Yoichi M. Ito, Yuji Kitaichi, Shin Nakagawa, Ichiro Kusumi

**Affiliations:** 1 Department of Psychiatry, Hokkaido University Graduate School of Medicine, Hokkaido, Japan; 2 Department of Psychiatry, Tokyo Medical University, Tokyo, Japan; 3 Department of Biostatistics, Hokkaido University Graduate School of Medicine, Sapporo, Hokkaido, Japan; Chiba Daigaku, JAPAN

## Abstract

**Background:**

Suicide rates are vastly higher in Japan than in many other countries, although the associations between affective temperaments and suicide-related ideations in the general adult population remain unclear. Therefore, we aimed to elucidate these associations in the present study.

**Methods:**

We analyzed data from 638 Japanese volunteers who completed both the Patient Health Questionnaire (PHQ-9) and the Temperament Evaluation of the Memphis, Pisa, Paris, and San Diego Auto-questionnaire (TEMPS-A). Participants were then divided into three groups based on PHQ-9 summary scores and responses to the suicide-related ideation item: non-depressive control group (NC; N = 469), depressive symptoms without suicide-related ideations group (non-SI; N = 135), and depressive symptoms with suicide-related ideations group (SI; N = 34). The depressive symptoms were defined for PHQ-9 summary scores ≥5, and the suicide-related ideations were defined for PHQ-9 #9 score ≥1. We then compared TEMPS-A scores among the groups using Kruskal-Wallis tests. Then the 95% confidence intervals of differences in TEMPS-A subscale scores between the NC and non-SI groups, or between NC and SI groups, were calculated.

**Results:**

Participants of the SI group exhibited significantly higher scores on the depressive, irritable, and anxious temperament subscales than those of the non-SI group. Similarly, women of the SI group exhibited significantly higher scores of the depressive and irritable temperament subscales than women of the non-SI group, while men of the SI group exhibited significantly higher depressive temperament scores than those of the non-SI group. Among all participants and only men, cyclothymic subscale scores were higher in those of the SI group than the non-SI group (not significant), although the 95% confidence intervals did not overlap.

**Limitations:**

The cross-sectional study design was the main limitation.

**Conclusions:**

Depressive, irritable, and anxious temperaments are significant risk factors for suicide-related ideations in the Japanese general adult population. Furthermore, irritable temperament in women and depressive temperament in men are associated with suicide-related ideations.

## Introduction

Suicide is a major public health concern in Japan, with overall rates approximately 60% higher than the global average. In 2007, the Japanese government initiated a nine-step plan to decrease suicide rates by 20% over the next ten years by encouraging the investigation of suicide risk factors, changing cultural attitudes toward suicide, and improving treatment for those who have attempted suicide. Although some progress has been made, the prediction and prevention of suicide remain a high priority in Japan.

Researchers have aimed to elucidate the various factors contributing to suicide, which are categorized as either proximal or distal[1]. Proximal factors such as a recent history of attempted suicide have been regarded as the most powerful risk factors for short-term suicide [2, 3]. However, more than half of completed suicides are associated with a first attempt [4], indicating that treatment for attempted suicide is insufficient for suicide prevention. Moreover, those who complete suicide are less likely to seek mental health services[5]. Thus, suicide prevention efforts may be greatly improved by elucidating factors contributing to suicide among the general population.

Personality traits are thought to be among the most important distal risk factors of suicide, and some previous studies have reported that specific personality characteristics are associated with suicidal behaviors as well as suicidal ideation[6, 7]. Previous studies have revealed associations between suicidal ideation and various characteristics such as hopelessness (Beck Hopelessness Scale), neuroticism (Eysenck Personality Questionnaire), and harm avoidance (Temperament and Character Inventory)[8–11]. Suicidal ideation is also significantly associated with suicide attempts[9, 12] and completions[10]. Furthermore, recent studies have indicated that affective temperaments are associated with both suicide attempts and suicidal ideation[13].

Developed by Akiskal and colleagues, the modern concept of affective temperaments consists of five basic temperament types: depressive, cyclothymic, hyperthymic, irritable, and anxious[14]. Individual variations in temperament are assessed using the Temperament Evaluation of Memphis, Pisa, Paris, and San Diego Autoquestionnaire (TEMPS-A) [14, 15], which has also been utilized in recent research regarding suicidal ideation and suicidal behaviors [13, 16–20]. Recently, several studies have revealed an association between affective temperaments and suicide-related behaviors among patients with mood disorders [16, 17, 21], noting that suicide attempts in this patient population were associated with depressive, irritable, anxious[22], and cyclothymic temperaments [22–24]. Another study involving patients with psychiatric conditions other than mood disorders indicated that suicide risk was associated with an irritable temperament [25].

Such findings suggest that affective temperaments play a significant role in the development of suicide-related behaviors in clinical populations. Although several studies have revealed an association between affective temperaments and suicide-related behaviors among individuals with mood disorders, relatively few have investigated such an association among the general population. One such study reported that a history of suicide attempts was significantly associated with depressive and cyclothymic temperaments in this population as well [20], while a recent study involving college students indicated that depressive, cyclothymic, and anxious temperaments were associated with suicidal ideation in both men and women [26]. A large-sample survey of the general population also suggested that anxious temperament is a strong risk factor for suicide attempts [18]. However, to our best knowledge, there is no study, which investigate the relationship between the affective temperaments and suicidal ideations among general adult population. Also, it is unclear the associations between gender and affective temperaments among the general adult population with suicidal ideations.

The aim of the present study was to investigate the association between affective temperament and suicidal ideation/thoughts of self-harm in the non-clinical general adult population. Gender differences of affective temperaments between participants with or without suicide-related ideations were also investigated in this study. To achieve this aim, we compared TEMPS-A subscale scores between participants with depression experiencing suicide-related ideations and those without suicide-related ideations.

## Materials and methods

### 1. Participants

The present study consisted of two survey periods (July 2011 to December 2011 and January 2014 to August 2014) involving 500 Japanese volunteers [27]and 853 Japanese volunteers [28], respectively. All participants, who had no history of psychiatric disease, were recruited from the non-clinical general adult population by flyers and word of mouth. We included data from 701 participants (286 from Survey 1 (57.2%) and 415 from Survey 2 (48.7%)) who completed both the Patient Health Questionnaire (PHQ-9) and the TEMPS-A. Sixty-one participants were further excluded from the Survey 2 dataset, as they had already participated in the first survey. Patient characteristics including gender, age, and education level were collected for all 640 remaining participants, who were then divided into three groups according to PHQ-9 scores. Participants with PHQ-9 summary scores ≤4 were categorized into the non-depressive control (NC) group. Participants with PHQ-9 summary scores >4 and PHQ-9 #9 scores of 0 were categorized into the “depressive symptoms without suicide-related ideations (non-SI)” group, while those with PHQ-9 summary scores >4 and PHQ-9 #9 scores ≥1 were categorized into the “depressive symptoms with suicide-related ideations (SI)” group. The PHQ-9 #9 scores ≥1 means that the participants had suicide-related ideations at least “several days” out of the week. We excluded two participants with PHQ-9 summary scores ≤4 and PHQ-9 #9 scores ≥1 to avoid bias associated with heterogeneous populations. A total of 638 (47.2%) participants were included in the final analysis.

Written informed consent was obtained from all participants. The present study was performed in accordance with the ethical standards established in the 1964 Declaration of Helsinki (amended in Fortaleza, October 2013) and approved by the institutional review board of Hokkaido University Hospital.

### 2. Measures

#### 2.1. PHQ-9

The PHQ was developed as a self-report version of the Primary Care Evaluation of Mental Disorders (PRIME-MD). It was designed for the criteria-based diagnosis of several mental disorders commonly observed in primary care [29]. The validity of the depression module (PHQ-9) of the PHQ for screening major depressive episodes has been confirmed in primary care, medical outpatient, and specialty medical settings. Two recent meta-analyses have reported reliable sensitivity (0.80 and 0.77, respectively) and specificity (0.92 and 0.94, respectively) for the PHQ-9 [30, 31]. These numbers correspond with the DSM-IV diagnosis of major depressive disorder or major depressive episodes in primary care clinics and non-psychiatric clinics.

In the present study, we used the Japanese version of the PHQ-9, which also has excellent validity in primary care [32] and in psychiatric settings [33]. The PHQ-9 summary score assesses the severity of depression, which is divided into five categories: 0–4, 5–9, 10–14, 15–19, 20 or greater [34]. In the present study, depressive symptoms were defined for PHQ-9 summary scores ≥5. After reviewing the data, when an individual responded that he or she had “Thoughts that you would be better off dead or of hurting yourself in some way” at least “several days” out of the week, he or she was considered to have “suicide-related ideations”. The meaning of the term “suicide-related ideations” includes ideas of both suicide and self-harm (Silverman et al., 2007). Thus, this term is coincident with a PHQ-9 #9 score ≥1.

#### 2.2. Temperament evaluation of the Memphis, Pisa, Paris, and San Diego auto-questionnaire (TEMPS-A)

The TEMPS-A is a dichotomous self- report questionnaire containing 109 items for men and 110 items for women used to assess variations in depressive, cyclothymic, hyperthymic, irritable, and anxious temperaments [14, 15]. The depressive, cyclothymic, hyperthymic, and irritable (20 for men) subscales consist of 21 items, while the anxious subscale consists of 26 items. The TEMPS-A Japanese version, which has shown reliability and validity (internal consistency) in a non-clinical sample of Japanese participants, was administered in the present study [35].

### 3. Statistical analyses

Patient characteristics were summarized using descriptive statistics and subjected to the Kruskal-Wallis test. *Post hoc* analysis was performed using Dunn’s test. Kruskal-Wallis and Dunn’s tests were also performed for the analysis of TEMPS-A subscale scores. The 95% confidence intervals of differences in TEMPS-A subscale scores between the NC and non-SI groups, or between NC and SI groups, were then calculated according to gender, following which the 95% confidence intervals of mean scores on the TEMPS-A were compared between those of the SI and non-SI groups. We calculated 95% confidence intervals for differences between two unpaired population means (Gardner and Altman, 1986). Differences were considered significant at *P*<0.05. SPSS version 21.0 (SPSS Inc., Japan) was used for all analyses.

## Results

### 1. Demographic data

The demographic data of the NC, non-SI, and SI groups are shown in Table 1. No significant differences in mean age were noted among the groups (P = 0.066). Women comprised 43.3% of the NC group (N = 469), 55.6% of the non-SI group (N = 135), and 64.7% of the SI group (N = 34), respectively. Kruskal-Wallis tests revealed significant differences in the distribution of gender among these three groups (P = 0.004). *Post hoc* analyses using Dunn's test revealed that this difference remained significant between the NC and non-SI groups (P = 0.012), and between the NC and SI groups (P = 0.016). Educational experiences were also significantly different among the groups (P = 0.009), and *post hoc* analysis revealed the NC group had significantly higher levels of education than the SI (P = 0.018), and non-SI (P = 0.026) groups. The distribution of marital status was significantly different among the three groups (P = 0.002), and *post hoc* analyses revealed that the marriage rate in NC group was significantly higher than that of the SI (P = 0.02) and non-SI (P = 0.003) groups. However, no significant difference was observed between the SI and non-SI groups (P = 0.508). Significant differences in PHQ-9 summary scores of these three groups were also observed. *Post hoc* analysis revealed that PHQ-9 summary scores of the SI and non-SI groups were significantly higher than those of the NC group (P<0.001 for each). However, no significant difference in PHQ-9 summary score was observed between the SI and non-SI groups (*P* = 0.307). No significant differences in demographic characteristics were observed between the SI and non-SI groups.

**Table 1:** Demographic data.

	NC	non-SI	SI	P
N	469	135	34	
Gender*				0.004
Female	203 (43.3%)	75 (55.6%)	22 (64.7%)	
Male	266 (56.7%)	60 (44.4%)	12 (35.3%)	
Age^†^, mean (SD)	42.9 (11.8)	40.9(12.2)	39.2(10.4)	0.066
Education*				0.009
Junior high school or High school	94 (20.4%)	40 (29.6%)	13 (38.2%)	
Junior college, College or Graduate school	367 (79.6%)	95 (70.4%)	21 (61.8%)	
Marital status*				0.002
Married	360 (76.8%)	87 (64.4%)	20 (58.8%)	
Unmarried	107 (22.9%)	48 (35.6%)	14 (41.2%)	
Past medical history*	93 (19.8%)	30 (22.2%)	9 (26.5%)	0.532
Family history of psychiatric disorders*	60 (12.8%)	18 (13.3%)	6 (17.6%)	0.727
PHQ-9 summary score^†^, mean (SD)	1.5(1.4)	7.1 (2.2)	12.7(5.5)	<0.001

*Kruskal-Wallis test

†ANOVA

Unknown cases of education (N = 8), and marital status (N = 2) were excluded.

NC, non-depressive control; non-SI, depressive symptoms without suicide-related ideations; SI, depressive symptoms with suicide-related ideations.

### 2. TEMPS-A scores

Kruskal-Wallis tests and *post hoc* analyses revealed that the depressive, cyclothymic, irritable, and anxious temperament scores of the SI and non-SI groups were significantly higher than those of the NC group (Table 2). Moreover, among all participants, the depressive, irritable, and anxious temperament scores of participants in the SI group were significantly higher than those of the non-SI group. Among female participants, the depressive and irritable temperament scores of participants in the SI group were significantly higher than those of women in the non-SI group. Among male participants, only the depressive temperament scores of men in the SI group were significantly higher than those of men in the non-SI group.

**Table 2:** Comparison of TEMPS-A scores among participants.

TEMPS-A	Gender	NC (1)	non-SI (2)	SI (3)	Statistics*	P	Post-hoc analyses†		
		Mean(SD)	Mean(SD)	Mean(SD)			(1) vs (2)	(1) vs (3)	(2) vs (3)
Depressive	All	1.32 (0.14)	1.42 (0.16)	1.54 (0.16)	81.6	<0.001	<0.001	<0.001	0.001
	Female	1.36 (0.14)	1.45 (0.16)	1.55 (0.16)	37.4	<0.001	<0.001	<0.001	0.019
	Male	1.29 (0.14)	1.38 (0.16)	1.53 (0.16)	36.5	<0.001	<0.001	<0.001	0.035
Cyclothymic	All	1.14 (0.15)	1.31 (0.22)	1.44 (0.27)	106.3	<0.001	<0.001	<0.001	0.075
	Female	1.15 (0.13)	1.35 (0.23)	1.43 (0.27)	63.2	<0.001	<0.001	<0.001	0.345
	Male	1.13 (0.15)	1.25 (0.18)	1.46 (0.30)	40.0	<0.001	<0.001	<0.001	0.158
Hyperthyic	All	1.28 (0.20)	1.22 (0.17)	1.23 (0.22)	4.7	0.006	0.005	0.066	0.789
	Female	1.24 (0.17)	1.22 (0.18)	1.19 (0.17)	2.7	0.257	-	-	-
	Male	1.31 (0.21)	1.23 (0.16)	1.31 (0.28)	7.6	0.022	0.006	0.728	0.358
Irritable	All	1.10 (0.13)	1.19 (0.16)	1.32 (0.23)	52.7	<0.001	<0.001	<0.001	0.017
	Female	1.10 (0.12)	1.21 (0.17)	1.34 (0.21)	54.8	<0.001	<0.001	<0.001	0.012
	Male	1.11 (0.13)	1.18 (0.15)	1.28 (0.28)	23.6	<0.001	<0.001	0.008	0.579
Anxious	All	1.14 (0.14)	1.28 (0.21)	1.40 (0.24)	72.2	<0.001	<0.001	<0.001	0.029
	Female	1.16 (0.15)	1.33 (0.22)	1.44 (0.23)	56.7	<0.001	<0.001	<0.001	0.097
	Male	1.12 (0.13)	1.22 (0.17)	1.32 (0.25)	26.8	<0.001	<0.001	0.001	0.297

* Kruskal-Walllis test

†Dunn test

NC, Non-depressive controls; non-SI, Depressive symptoms without suicide-related ideations; SI, Depressive symptoms with suicide-related ideations

**Fig 1:**
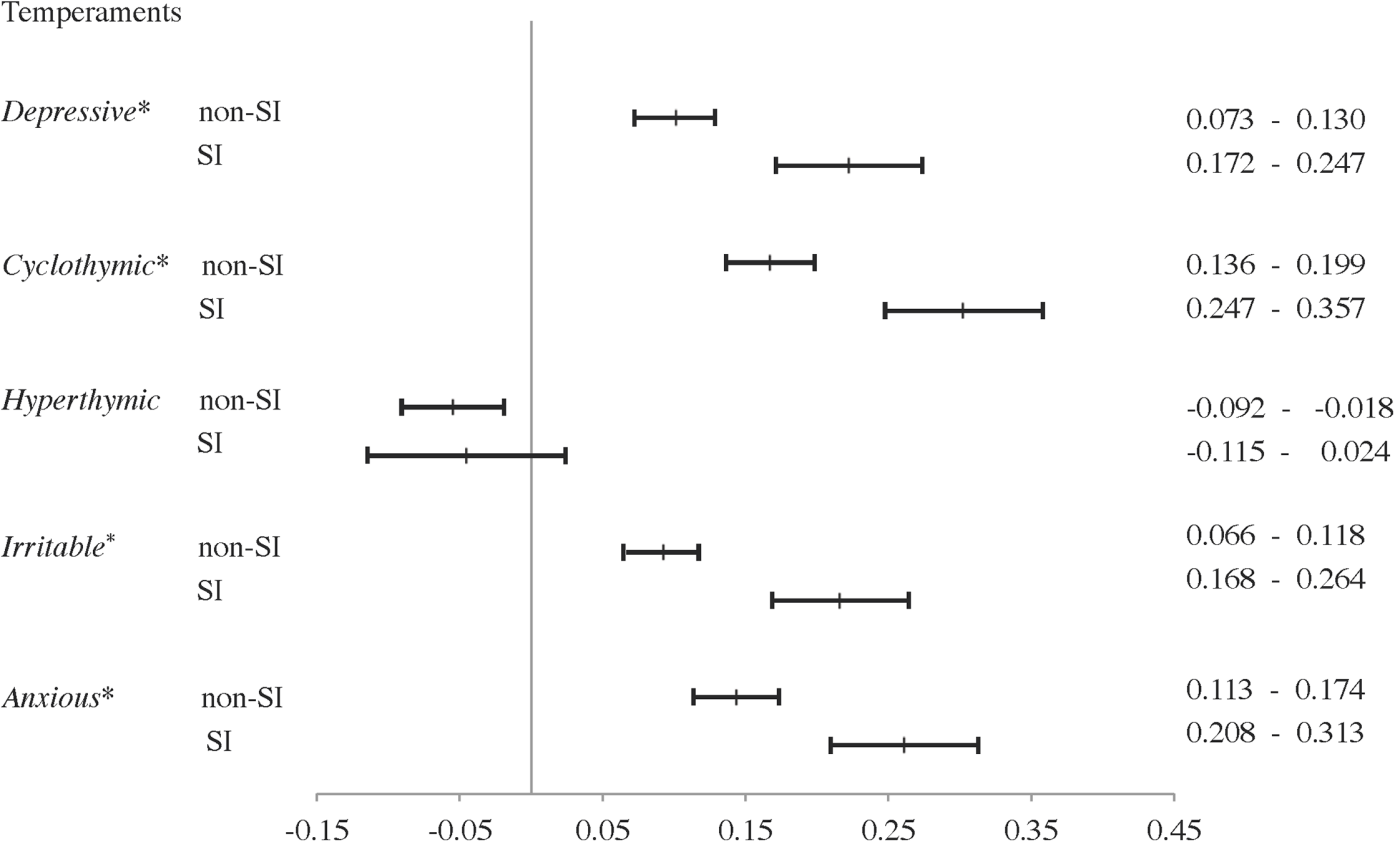
Comparison of TEMPS-A between participants with or without suicide-related ideations on TEMPS-A scores via 95% confidence intervals. SI, depressive symptoms with suicide-related ideations; non-SI, depressive symptoms without suicide-related ideations; TEMPS-A, Temperament Evaluation of Memphis, Pisa, Paris, and San Diego Auto-questionnaire. * 95% Confidence Intervals of non-SI group did not overlap with those of the SI group.

**Fig 2:**
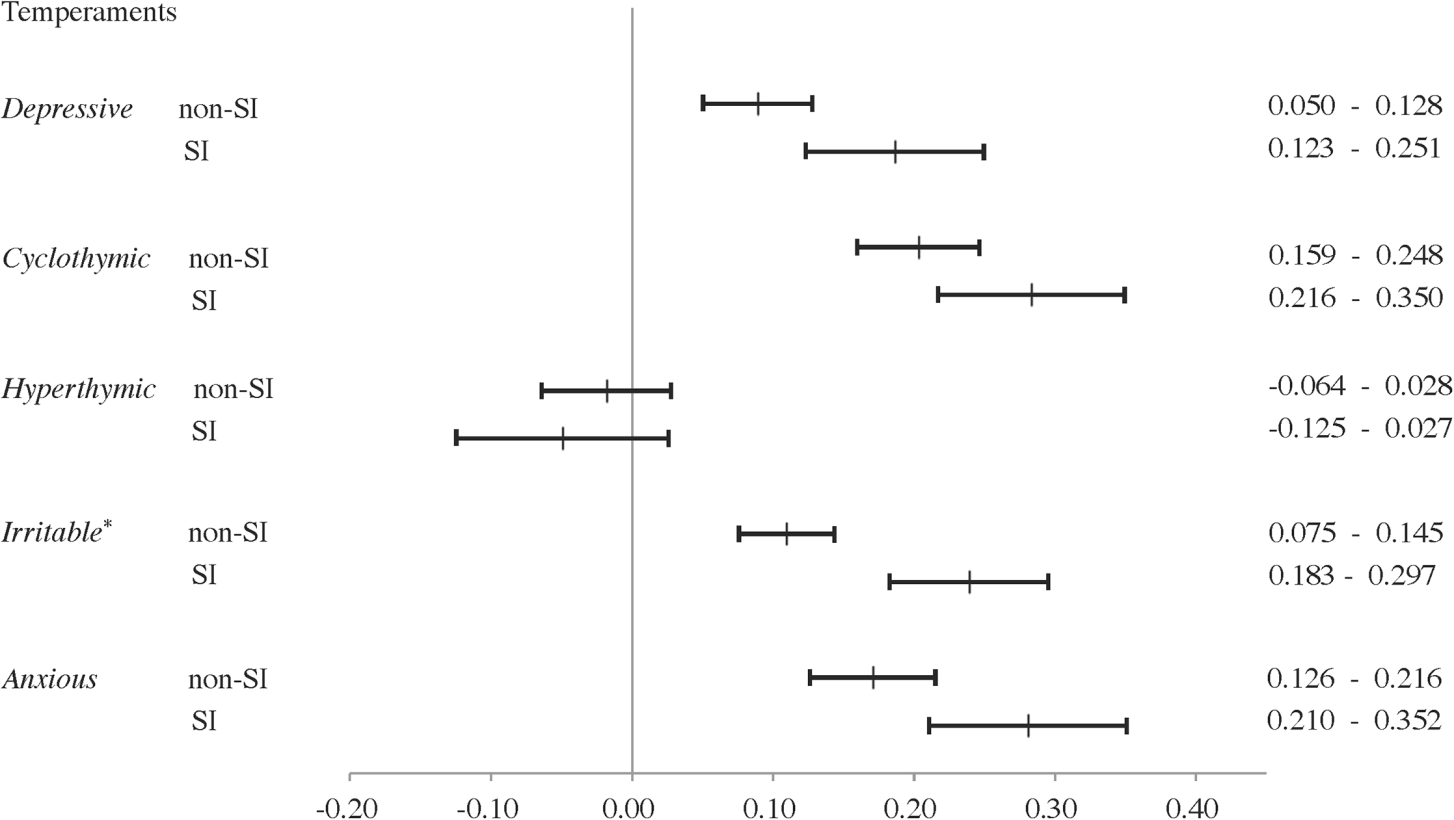
Comparison of TEMPS-A scores between women with or without suicide-related ideations on TEMPS-A scores via 95% confidence intervals. SI, depressive symptoms with suicide-related ideations; non-SI, depressive symptoms without suicide-related ideations; TEMPS-A, Temperament Evaluation of Memphis, Pisa, Paris, and San Diego Auto-questionnaire. * 95% Confidence Intervals of non-SI group did not overlap with those of the SI group.

**Fig 3:**
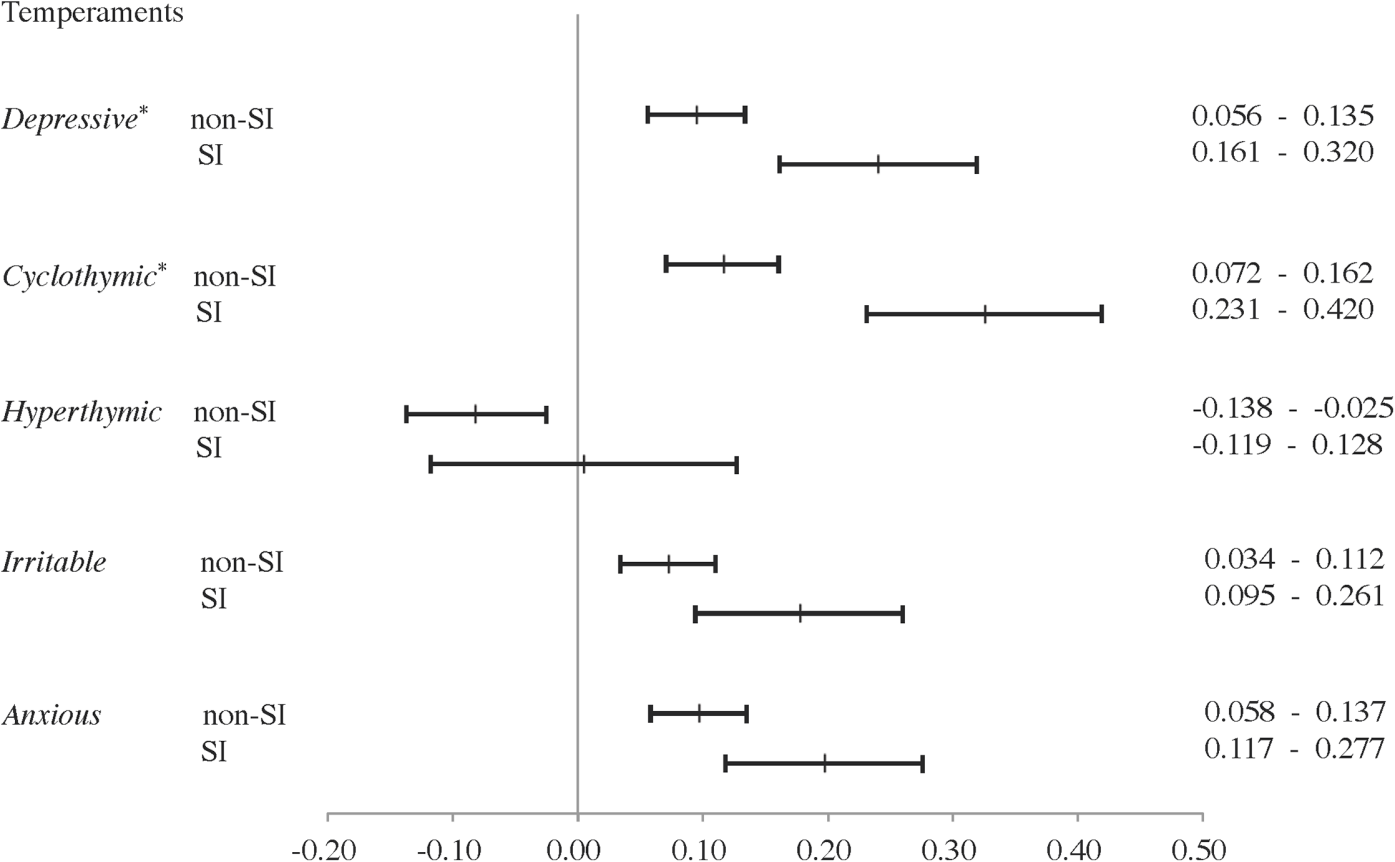
Comparison between depressive men with or without suicide-related ideations on TEMPS-A scores by 95% confidence intervals. SI, depressive symptoms with suicide-related ideations; non-SI, depressive symptoms without suicide-related ideations; TEMPS-A, Temperament Evaluation of Memphis, Pisa, Paris, and San Diego Auto-questionnaire. * 95% Confidence Intervals of non-SI group did not overlap with those of the SI group.

Among all participants, the 95% confidence intervals of mean depressive, cyclothymic, irritable, and anxious temperament scores in the SI group were higher than those in the non-SI group (Fig 1). Among female participants, the 95% confidence intervals for mean score differences in irritable temperament were higher for women of the SI group than for those of the non-SI group (Fig 2). Among male participants, the 95% confidence intervals for mean score differences in depressive and cyclothymic temperaments were higher for men of the SI group than those of the non-SI group (Fig 3).

The results of Kruskal-Wallis tests and *post hoc* tests were consistent with the results of the 95% confidence intervals of mean score on the depressive, irritable, and anxious temperaments in all participants, the irritable temperament in female participants, and the depressive temperament in male participants. Kruskal-Wallis tests and *post hoc* tests revealed that female participants of the SI group had significantly higher depressive temperament scores than those of the non-SI group, although the 95% confidence intervals overlapped. These analyses also revealed that, among all participants and only men, cyclothymic subscale scores were higher in those of the SI group than the non-SI group (not significant), although the 95% confidence intervals did not overlap.

## Discussion

The present study revealed that adult participants of the general population with depressive symptoms experiencing suicide-related ideations (i.e., ideas of suicide or self-harm) exhibited higher scores on the TEMPS-A depressive, irritable, and anxious temperament subscales than those without suicide-related ideations. Furthermore, women with suicide-related ideations exhibited higher scores on the irritable temperament subscale than those without suicide-related ideations. In contrast, men with suicide-related ideations exhibited higher scores on the depressive temperament subscale than those without suicide-related ideations.

These results suggest that depressive temperament contributes significantly to the incidence of suicide-related ideations in individuals of the general adult population with sub-clinical depressive symptoms, consisting with the findings of previous studies regarding attempted suicide. In a previous cross-sectional study of public school students (n = 1,713), depressive temperament was significantly associated with self-harm in both genders [36]. In another previous study of 150 nonviolent suicide attempters, scores for the depressive, cyclothymic, irritable, and anxious temperament subscales were significantly associated with suicide attempts, and the odds ratios for dominant depressive temperament (mean+ 2SD or above) was significantly high [22]. Furthermore, in a cross-sectional study involving healthy college students (n = 1,381), the TEMPS-M depressive, cyclothymic, and anxious temperament scores were significantly associated with suicidal ideations, and depressive temperament had the highest odds ratio as a predictor of lifetime suicidal ideations among all participants [26]. These findings suggest that depressive temperament plays an important role in the development of suicide-related ideations. Individuals with depressive temperament have a tendency towards lowered mood, and the primary cognitive features associated with depressive temperament include low self-esteem and self-criticism [14]. Previous studies have indicated that self-esteem is negatively correlated with suicide rates at the national level, especially among Japanese individuals, in whom the lowest levels of self-esteem were observed [37]. Previously, we observed significant relationships between suicide risk and low self-esteem among Japanese university students [38]. The present study suggests that depressive temperament as measured by the TEMPS-A is among the most important risk factors for suicide-related ideations in the general adult population, especially in men.

In the present study, the irritable temperament was significantly associated with suicide-related ideations in women. The irritable temperament is thought to be a strong predictor of suicidal behavior in clinical populations [13, 22, 25, 39], while the combination of irritable temperament and major depressive episodes results in a mixed depressive state, which is thought to be one of the most important risk factors for suicide [40, 41]. Several previous studies have revealed that the relationship between irritable temperament and suicide-related ideations, however among the general female population remains unclear [20, 26, 42]. Furthermore, the reason for this gender difference remains unclear as well.

In the present study, no significant differences in cyclothymic temperament were observed among participants with and without suicide-related ideations, although the 95% confidence intervals of differences in cyclothymic temperament between the NC and non-SI/SI groups did not overlap. These findings suggest that cyclothymic temperament is related to depressive symptoms, but not to suicide-related ideations. Recent reports have suggested that cyclothymic temperament plays a significant role in the development of suicidal behavior among patients with mood disorders [39]. In a previous study, patients with cyclothymic temperament and bipolar Ⅱ disorder experienced greater numbers of lifetime suicide attempts and suicide-related hospitalizations than those without cyclothymic temperament [43]. Additional studies have reported that cyclothymic temperament is significantly associated with suicide attempts in clinical populations [22–24]. A recent study reported that patients with mood disorders and cyclothymic temperament have a higher risk of suicide than those without cyclothymic temperament [17]. Moreover, cyclothymic temperament has been associated with psychiatric conditions other than mood disorders, such as borderline personality disorder, alcohol related disorders, and bulimia nervosa [44], which are known as high risk factors for suicidal behavior [45, 46]. Previous studies have also suggested that cyclothymic temperament was associated with both suicide attempts and suicidal ideation among children and adolescents with mood disorders [24]. These findings are also consistent with the results of a previous study involving patients in primary care settings, which reported that cyclothymic and depressive temperaments were significantly associated with personal history of suicide attempts [20]. In a study of non-clinical college students, cyclothymic, depressive, and anxious temperaments were significantly associated with suicidal ideation [26]. In a review of affective temperaments in the general population, Vazquez et al. revealed that cyclothymic temperament did not correlate with depressive temperament [42]. These findings suggest that the effect of cyclothymic temperament on suicide-related ideations differs from that of depressive temperament.

We also observed that anxious temperament was associated with suicide-related ideations among the general population in the present study. Previous studies have also reported that anxious temperament is significantly associated with suicide risk among psychiatric inpatients [25], with nonviolent suicide attempts [22], and with family history of suicide attempts [20]. In a recent large-scale study involving participants from among the general population, the TEMPS-A anxious temperament was identified as a strong risk factor for suicide attempts [18].

However, hyperthymic temperament did not significantly differ between participants with and without suicide-related ideations in the present study, consistent in part with the results of several previous studies, which suggested that hyperthymic temperament may be a protective factor against both suicide risk and hopelessness [13, 39].

The cross-sectional study design is a main limitation of the present study. Thus, additional prospective longitudinal studies are required in order to more fully elucidate factors predictive of suicide-related ideations. Furthermore, we observed some differences in the distribution of gender and educational experience among the three groups, which may have influenced the outcome of our study. Since we excluded non-depressive participants with suicide-related ideations, we could not analyze these populations. In the future, we need another study for investigating TEMPS-A of non-depressive populations with suicide-related ideations. Additionally, since the number of male participants with suicide-related ideations was small, the results of the Kruskal-Wallis tests were not entirely consistent with the results of the statistical estimations, which were described as 95% confidence intervals. The use of the PHQ-9 to evaluate suicide-related ideations represents another major limitation of the present study, as thoughts of suicide generally differ from thoughts of self-harm in a number of respects. Hence, a specific scale for the evaluation of suicidal ideation should be utilized in future studies. The results of the present study could not be generalized to other populations in different countries, because both universal and cultural specificity of affective temperaments were suggested in the previous study [42].

## Conclusions

Our findings indicate that the TEMPS-A depressive, irritable, and anxious temperaments are significant risk factors for suicide-related ideations in the Japanese non-clinical general adult population. We further observed that the TEMPS-A depressive temperament is a specific risk factor for suicide-related ideations among men, while the TEMPS-A irritable temperament is specific risk factor for suicide-related ideations among women.

## References

[1] Hawton K, Saunders KE. Psychiatric service development and suicide. Lancet. 2009;373(9658):99–100. doi: 10.1016/S0140-6736(08)61871-1 . 19097637 10.1016/S0140-6736(08)61871-1

[2] Oquendo MA, Currier D, Mann JJ. Prospective studies of suicidal behavior in major depressive and bipolar disorders: what is the evidence for predictive risk factors? Acta Psychiatr Scand. 2006;114(3):151–8. doi: 10.1111/j.1600-0447.2006.00829.x . 16889585 10.1111/j.1600-0447.2006.00829.x

[3] Owens D, Horrocks J, House A. Fatal and non-fatal repetition of self-harm. Systematic review. Br J Psychiatry. 2002;181:193–9. . 12204922 10.1192/bjp.181.3.193

[4] Isometsa ET, Lonnqvist JK. Suicide attempts preceding completed suicide. Br J Psychiatry. 1998;173:531–5. . 9926085 10.1192/bjp.173.6.531

[5] Paris J. Predicting and preventing suicide: do we know enough to do either? Harv Rev Psychiatry. 2006;14(5):233–40. doi: 10.1080/10673220600968662 . 16990168 10.1080/10673220600968662

[6] Brezo J, Paris J, Turecki G. Personality traits as correlates of suicidal ideation, suicide attempts, and suicide completions: a systematic review. Acta Psychiatr Scand. 2006;113(3):180–206. doi: 10.1111/j.1600-0447.2005.00702.x . 16466403 10.1111/j.1600-0447.2005.00702.x

[7] Mitsui N, Asakura S, Inoue T, Shimizu Y, Fujii Y, Kako Y, et al Temperament and character profiles of Japanese university student suicide completers. Compr Psychiatry. 2013;54(5):556–61. doi: 10.1016/j.comppsych.2012.11.002 . 23246072 10.1016/j.comppsych.2012.11.002

[8] Beevers CG, Miller IW. Perfectionism, cognitive bias, and hopelessness as prospective predictors of suicidal ideation. Suicide Life Threat Behav. 2004;34(2):126–37. doi: 10.1521/suli.34.2.126.32791 . 15191269 10.1521/suli.34.2.126.32791

[9] Conrad R, Walz F, Geiser F, Imbierowicz K, Liedtke R, Wegener I. Temperament and character personality profile in relation to suicidal ideation and suicide attempts in major depressed patients. Psychiatry Res. 2009;170(2–3):212–7. S0165-1781(08)00347-8 [pii] doi: 10.1016/j.psychres.2008.09.008 . 19897251 10.1016/j.psychres.2008.09.008

[10] Mitsui N, Asakura S, Shimizu Y, Fujii Y, Kako Y, Tanaka T, et al Temperament and character profiles of Japanese university students with depressive episodes and ideas of suicide or self-harm: A PHQ-9 screening study. Compr Psychiatry. 2013;54(8):1215–21. doi: 10.1016/j.comppsych.2013.05.014 . 23849616 10.1016/j.comppsych.2013.05.014

[11] Statham DJ, Heath AC, Madden PA, Bucholz KK, Bierut L, Dinwiddie SH, et al Suicidal behaviour: an epidemiological and genetic study. Psychol Med. 1998;28(4):839–55. . 9723140 10.1017/s0033291798006916

[12] Calati R, Giegling I, Rujescu D, Hartmann AM, Moller HJ, De Ronchi D, et al Temperament and character of suicide attempters. J Psychiatr Res. 2008;42(11):938–45. doi: 10.1016/j.jpsychires.2007.10.006 . 18054960 10.1016/j.jpsychires.2007.10.006

[13] Rihmer Z, Akiskal KK, Rihmer A, Akiskal HS. Current research on affective temperaments. Curr Opin Psychiatry. 2010;23(1):12–8. doi: 10.1097/YCO.0b013e32833299d4 . 19809321 10.1097/YCO.0b013e32833299d4

[14] Akiskal HS, Akiskal KK, Haykal RF, Manning JS, Connor PD. TEMPS-A: progress towards validation of a self-rated clinical version of the Temperament Evaluation of the Memphis, Pisa, Paris, and San Diego Autoquestionnaire. J Affect Disord. 2005;85(1–2):3–16. doi: 10.1016/j.jad.2004.12.001 . 15780671 10.1016/j.jad.2004.12.001

[15] Akiskal HS. Toward a definition of generalized anxiety disorder as an anxious temperament type. Acta Psychiatr Scand Suppl. 1998;393:66–73. . 9777050 10.1111/j.1600-0447.1998.tb05969.x

[16] Baldessarini RJ, Vazquez GH, Tondo L. Affective temperaments and suicidal ideation and behavior in mood and anxiety disorder patients. J Affect Disord. 2016;198:78–82. doi: 10.1016/j.jad.2016.03.002 . 27011363 10.1016/j.jad.2016.03.002

[17] Innamorati M, Rihmer Z, Akiskal H, Gonda X, Erbuto D, Belvederi Murri M, et al Cyclothymic temperament rather than polarity is associated with hopelessness and suicidality in hospitalized patients with mood disorders. J Affect Disord. 2015;170:161–5. doi: 10.1016/j.jad.2014.08.042 . 25240844 10.1016/j.jad.2014.08.042

[18] Karam EG, Itani L, Fayyad J, Hantouche E, Karam A, Mneimneh Z, et al Temperament and suicide: A national study. J Affect Disord. 2015;184:123–8. doi: 10.1016/j.jad.2015.05.047 . 26080077 10.1016/j.jad.2015.05.047

[19] Rihmer Z, Gonda X. Predisposition for self-destruction? Affective temperaments as a suicide risk factor in patients with mood disorders. Crisis. 2012;33(6):309–12. doi: 10.1027/0227-5910/a000192 . 23165107 10.1027/0227-5910/a000192

[20] Rihmer Z, Gonda X, Torzsa P, Kalabay L, Akiskal HS, Eory A. Affective temperament, history of suicide attempt and family history of suicide in general practice patients. J Affect Disord. 2013;149(1–3):350–4. doi: 10.1016/j.jad.2013.02.010 . 23477849 10.1016/j.jad.2013.02.010

[21] Pompili M, Innamorati M, Raja M, Falcone I, Ducci G, Angeletti G, et al Suicide risk in depression and bipolar disorder: Do impulsiveness-aggressiveness and pharmacotherapy predict suicidal intent? Neuropsychiatric disease and treatment. 2008;4(1):247–55. . 18728807 10.2147/ndt.s2192 PMC2515901

[22] Rihmer A, Rozsa S, Rihmer Z, Gonda X, Akiskal KK, Akiskal HS. Affective temperaments, as measured by TEMPS-A, among nonviolent suicide attempters. J Affect Disord. 2009;116(1–2):18–22. doi: 10.1016/j.jad.2008.10.024 . 19036456 10.1016/j.jad.2008.10.024

[23] Azorin JM, Kaladjian A, Adida M, Hantouche E, Hameg A, Lancrenon S, et al Risk factors associated with lifetime suicide attempts in bipolar I patients: findings from a French National Cohort. Compr Psychiatry. 2009;50(2):115–20. doi: 10.1016/j.comppsych.2008.07.004 . 19216887 10.1016/j.comppsych.2008.07.004

[24] Kochman FJ, Hantouche EG, Ferrari P, Lancrenon S, Bayart D, Akiskal HS. Cyclothymic temperament as a prospective predictor of bipolarity and suicidality in children and adolescents with major depressive disorder. J Affect Disord. 2005;85(1–2):181–9. doi: 10.1016/j.jad.2003.09.009 . 15780688 10.1016/j.jad.2003.09.009

[25] Pompili M, Rihmer Z, Akiskal HS, Innamorati M, Iliceto P, Akiskal KK, et al Temperament and personality dimensions in suicidal and nonsuicidal psychiatric inpatients. Psychopathology. 2008;41(5):313–21. doi: 10.1159/000146069 . 18635934 10.1159/000146069

[26] Skala K, Kapusta ND, Schlaff G, Unseld M, Erfurth A, Lesch OM, et al Suicidal ideation and temperament: an investigation among college students. J Affect Disord. 2012;141(2–3):399–405. doi: 10.1016/j.jad.2012.03.010 . 22475473 10.1016/j.jad.2012.03.010

[27] Nakai Y, Inoue T, Toda H, Toyomaki A, Nakato Y, Nakagawa S, et al The influence of childhood abuse, adult stressful life events and temperaments on depressive symptoms in the nonclinical general adult population. J Affect Disord. 2014;158:101–7. doi: 10.1016/j.jad.2014.02.004 . 24655773 10.1016/j.jad.2014.02.004

[28] Nakai Y, Inoue T, Chen C, Toda H, Toyomaki A, Nakato Y, et al The moderator effects of affective temperaments, childhood abuse and adult stressful life events on depressive symptoms in the nonclinical general adult population. J Affect Disord. 2015;187:203–10. doi: 10.1016/j.jad.2015.08.011 . 26342173 10.1016/j.jad.2015.08.011

[29] Spitzer RL, Kroenke K, Williams JB. Validation and utility of a self-report version of PRIME-MD: the PHQ primary care study. Primary Care Evaluation of Mental Disorders. Patient Health Questionnaire. JAMA. 1999;282(18):1737–44. . 10568646 10.1001/jama.282.18.1737

[30] Gilbody S, Richards D, Brealey S, Hewitt C. Screening for depression in medical settings with the Patient Health Questionnaire (PHQ): a diagnostic meta-analysis. J Gen Intern Med. 2007;22(11):1596–602. doi: 10.1007/s11606-007-0333-y . 17874169 10.1007/s11606-007-0333-y PMC2219806

[31] Wittkampf KA, Naeije L, Schene AH, Huyser J, van Weert HC. Diagnostic accuracy of the mood module of the Patient Health Questionnaire: a systematic review. Gen Hosp Psychiatry. 2007;29(5):388–95. doi: 10.1016/j.genhosppsych.2007.06.004 . 17888804 10.1016/j.genhosppsych.2007.06.004

[32] Muramatsu K, Miyaoka H, Kamijima K, Muramatsu Y, Yoshida M, Otsubo T, et al The patient health questionnaire, Japanese version: validity according to the mini-international neuropsychiatric interview-plus. Psychol Rep. 2007;101(3 Pt 1):952–60. doi: 10.2466/pr0.101.3.952-960 18232454 10.2466/pr0.101.3.952-960

[33] Inoue T, Tanaka T, Nakagawa S, Nakato Y, Kameyama R, Boku S, et al Utility and limitations of PHQ-9 in a clinic specializing in psychiatric care. BMC psychiatry. 2012;12:73 doi: 10.1186/1471-244X-12-73 . 22759625 10.1186/1471-244X-12-73 PMC3416649

[34] Kroenke K, Spitzer RL, Williams JB. The PHQ-9: validity of a brief depression severity measure. J Gen Intern Med. 2001;16(9):606–13. doi: 10.1046/j.1525-1497.2001.016009606.x 11556941 10.1046/j.1525-1497.2001.016009606.x PMC1495268

[35] Matsumoto S, Akiyama T, Tsuda H, Miyake Y, Kawamura Y, Noda T, et al Reliability and validity of TEMPS-A in a Japanese non-clinical population: application to unipolar and bipolar depressives. J Affect Disord. 2005;85(1–2):85–92. doi: 10.1016/j.jad.2003.10.001 . 15780679 10.1016/j.jad.2003.10.001

[36] Guerreiro DF, Sampaio D, Rihmer Z, Gonda X, Figueira ML. Affective temperaments and self-harm in adolescents: a cross-sectional study from a community sample. J Affect Disord. 2013;151(3):891–8. doi: 10.1016/j.jad.2013.07.034 . 24035491 10.1016/j.jad.2013.07.034

[37] Chatard A, Selimbegovic L, Konan PND. Self-esteem and suicide rates in 55 nations. European Journal of Personality. 2009;.23(1):pp. doi: 10.1002/per.701 .

[38] Mitsui N, Asakura S, Shimizu Y, Fujii Y, Toyomaki A, Kako Y, et al The association between suicide risk and self-esteem in Japanese university students with major depressive episodes of major depressive disorder. Neuropsychiatric disease and treatment. 2014;10:811–6. doi: 10.2147/NDT.S59349 . 24868158 10.2147/NDT.S59349 PMC4031242

[39] Pompili M, Rihmer Z, Innamorati M, Lester D, Girardi P, Tatarelli R. Assessment and treatment of suicide risk in bipolar disorders. Expert Rev Neurother. 2009;9(1):109–36. doi: 10.1586/14737175.9.1.109 . 19102673 10.1586/14737175.9.1.109

[40] Akiskal HS, Benazzi F, Perugi G, Rihmer Z. Agitated "unipolar" depression re-conceptualized as a depressive mixed state: implications for the antidepressant-suicide controversy. J Affect Disord. 2005;85(3):245–58. doi: 10.1016/j.jad.2004.12.004 . 15780694 10.1016/j.jad.2004.12.004

[41] Balazs J, Benazzi F, Rihmer Z, Rihmer A, Akiskal KK, Akiskal HS. The close link between suicide attempts and mixed (bipolar) depression: implications for suicide prevention. J Affect Disord. 2006;91(2–3):133–8. doi: 10.1016/j.jad.2005.12.049 . 16458364 10.1016/j.jad.2005.12.049

[42] Vazquez GH, Tondo L, Mazzarini L, Gonda X. Affective temperaments in general population: a review and combined analysis from national studies. J Affect Disord. 2012;139(1):18–22. doi: 10.1016/j.jad.2011.06.032 . 21774989 10.1016/j.jad.2011.06.032

[43] Akiskal HS, Hantouche EG, Allilaire JF. Bipolar II with and without cyclothymic temperament: "dark" and "sunny" expressions of soft bipolarity. J Affect Disord. 2003;73(1–2):49–57. . 12507737 10.1016/s0165-0327(02)00320-8

[44] Perugi G, Toni C, Travierso MC, Akiskal HS. The role of cyclothymia in atypical depression: toward a data-based reconceptualization of the borderline-bipolar II connection. J Affect Disord. 2003;73(1–2):87–98. . 12507741 10.1016/s0165-0327(02)00329-4

[45] Cavanagh JT, Carson AJ, Sharpe M, Lawrie SM. Psychological autopsy studies of suicide: a systematic review. Psychol Med. 2003;33(3):395–405. . 12701661 10.1017/s0033291702006943

[46] Svirko E, Hawton K. Self-injurious behavior and eating disorders: the extent and nature of the association. Suicide Life Threat Behav. 2007;37(4):409–21. doi: 10.1521/suli.2007.37.4.409 . 17896881 10.1521/suli.2007.37.4.409

